# Supplementary material for: Simulations of scenarios for urban household water and energy consumption
Source: PLoS One. 2021 Apr 7;16(4):e0249781. doi: 10.1371/journal.pone.0249781 (PMC8026023; doi:10.1371/journal.pone.0249781)
Supplement: S1 File — (DOCX) [file pone.0249781.s001.docx]

**Supplementary material**

**Simulations of scenarios for household water and energy consumption**

Marco Casazza^1,2^, Jingyan Xue^3^, Shupan Du^3^, Gengyuan Liu^3^, Sergio Ulgiati^1,3^

1. Department of Sciences and Technologies, University of Napoli ‘Parthenope’, Centro Direzionale, Isola C4, 80143, Napoli (Italy)
2. Interdepartmental Research Centre on Urban and Event Studies (OMERO), University of Torino, Lungo Dora Siena 100 A, 10153, Torino (Italy)
3. School of Environment, Beijing Normal University, Beijing (China)

Corresponding author: Marco Casazza: [marco.casazza@uniparthenope.it](mailto:marco.casazza@uniparthenope.it)

**S1. Household FEW nexus structure**

Figure 1 shows the causal loop diagrams of the three subsystems (e.g., food, energy and water) and then merges them into the food-energy-water (FEW) system. A causal loop diagram represents the relationships among different variables, which are connected by arrows and represent a structural cause-effect relationship [1]. The direction of the causal link is represented by the arrowhead, while the polarity symbol (+ or -) identifies the type of relationship (positive or negative). In particular, a positive link indicates that a variable (the one close to the arrowhead) exhibits changes in value that are in the same direction as a second variable. The opposite is true for a negative link.

The causal loop diagrams shown in Figure 1S as well as the system simulations were generated using Vensim software produced by Ventana systems inc. The energy and water subsystems display a similar structure. Socioeconomic factors, such as age, sex, weather and income, are not considered in the simulations. Several feedback loops are employed in model construction. Household energy/water consumption is positively related to the household comfort standard. Thus, energy/water saving behaviours will be difficult engage in because these choices may impact perceived comfort levels. On the other hand, lower prices for efficient appliances would be beneficial for promoting technology measures. More households will retrofit higher-level appliances if the devices are price friendly. This, in turn, would increase the market share of efficient appliances. According to the law of supply and demand, the model assumes that when more efficient devices are needed, more such devices will be produced. Then, the device costs should become lower.

| 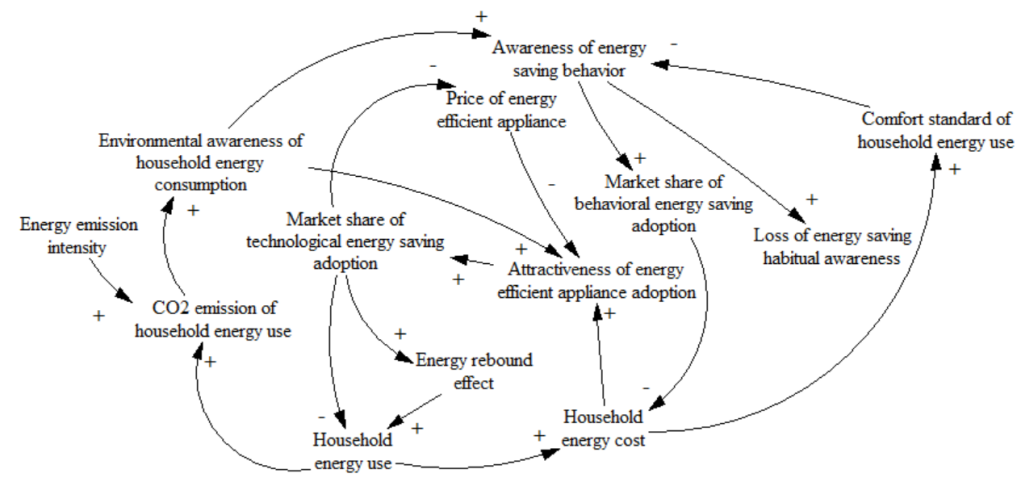 |
| --- |
| **(a)** |
| 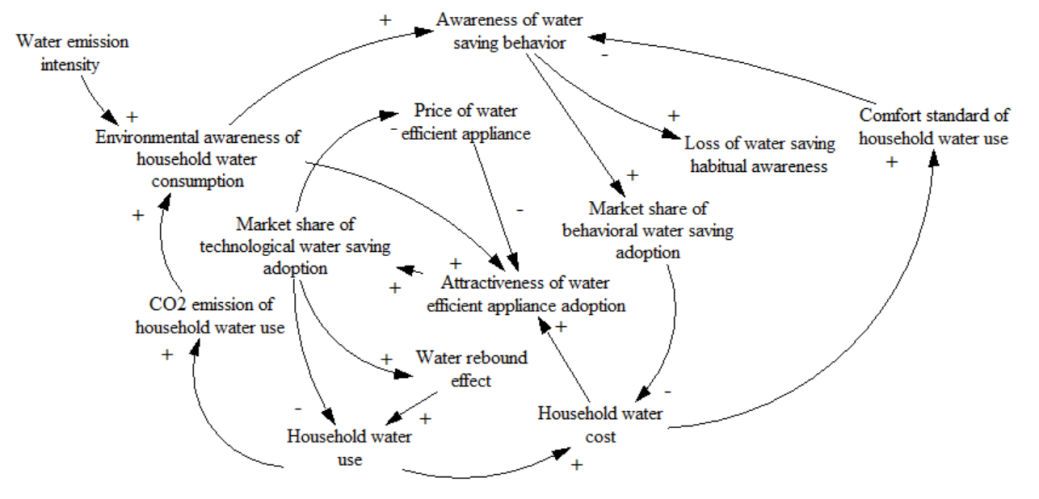 |
| **(b)** |
| 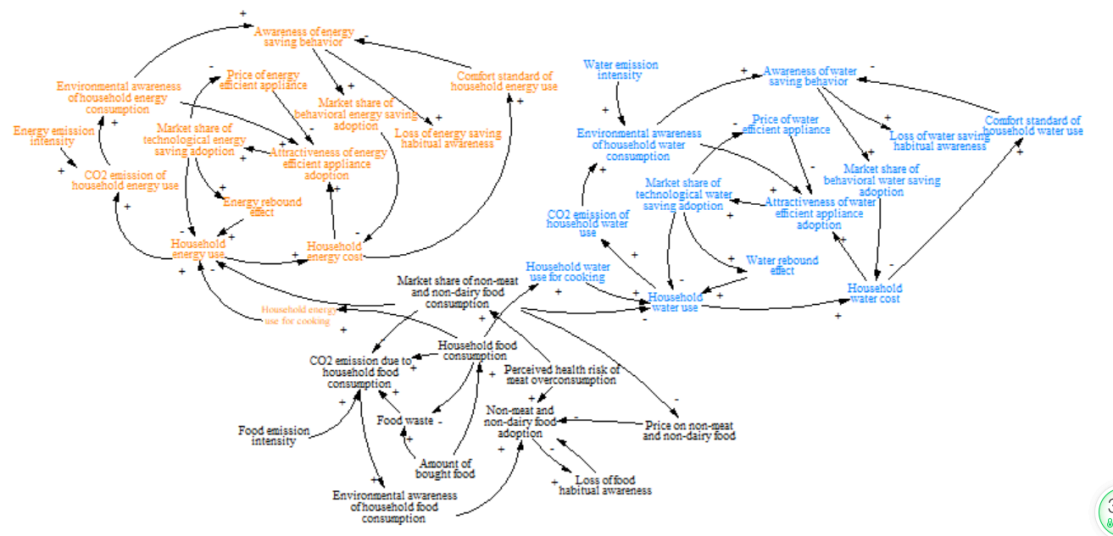 |
| **©** |

**Figure 1S.** Causal loop diagrams for household energy (a), water (b) and food (c) subsystems. The connections among the three subsystems, which generate a household FEW system, are represented in (d).

Four main balancing feedback loops are included in the model design. First, the full adoption of resource-saving behaviours depends on the time that is required to adopt such resource-saving behaviours and to what extent the behaviours can be ignored once they are fully adopted, i.e., behavioural resistance [2]. Moreover, the maintenance of resource curtailment habits is 1 year. This means that the awareness of energy/water-saving behaviours will gradually be lost without other measures. The longer it takes to form a habit, the more favourable it will be for the maintenance of energy-saving habits [3]. Household energy consumption costs are positively related to household energy use. An increase in energy costs will improve the adoption rate of energy-saving behaviours and technologies to reduce the related cumulative energy consumption. The attractiveness of efficient appliance adoption and saving behaviour adoption is positively related to household environmental concerns, which are influenced by greenhouse gases (GHG) emissions. More water/energy consumption leads to higher GHG emissions. Therefore, more technologies or curtailment behaviours will be adopted by households. The fourth balancing feedback is related to the rebound effect for which a specific element diminishes the effectiveness of technological measures. Higher adoption of technological measures will lead to greater rebound effects. This implies that households will increase their frequency or duration of appliance utilization after device upgrading.

Being different from the energy/water subsystem, the food subsystem is mainly influenced by the price of food, health perception of food types and household environmental concerns about food consumption. Food prices have a negative impact on residents’ purchasing intentions. People’s perceptions of meat and dairy foods also have effects on residents’ purchasing choices. Households may prefer nonmeat and dairy foods to avoid risks from obesity or cancer. In addition, the level of environmental concern regarding greenhouse gas emissions is associated with food choices. Meat and dairy foods are always accompanied by greater carbon footprints. The higher the environmental concern of households due to their food consumption is, the higher their willingness to adopt nonmeat and nondairy food consumption behaviours.

Food, energy and water subsystems are closely interlinked. Changes in any one of them will influence the resource consumption of the others. Energy and water are interconnected. Both heating water and running water appliances, such as washing machines or dryers, require energy. Water and energy are also needed for preparing cooked food in households. In addition, the fractions of meat and dairy in household dietary patterns will have an effect on the energy and water used for cooking. The water and energy consumption for meat and dairy-based food cooking is approximately three times that for nonmeat and nondairy cooking [4].

Figure 2S shows the structure and parameters that are related to the two subsystems that are included in the simulator: the food-water nexus subsystem and food-energy nexus subsystem. These subcomponents are merged into the household FEW nexus system.

|  |
| --- |
| **(a)** |
|  |
| **(b)** |
|  |
| **(c)** |

**Figure 2S.** Household FEW nexus model: (a) Household food-water nexus subsystem, (b) Household food-energy nexus, and (c) Household FEW nexus

**S2. Simulation scenarios**

| **Policy** | **Description** | **No. (Policy scenario)** | **Parameter adjustment** |
| --- | --- | --- | --- |
| Mass information provision to households on the benefit of sustainable resource-saving technology | Current situation for resource-saving appliances in Napoli. There is still a large potential for the promotion of resource-saving appliances in Napoli | BAU | perceived fraction of water saving given water tech adoption=0.5; perceived fraction of energy saving from energy tech adoption=0.5 |
|  | Provide more knowledge to adopt resource-saving appliances through newspaper, TV advertisements, and posters. (60% increase in implementation strength) | A1 | perceived fraction of water saving given water tech adoption=0.8; perceived fraction of energy saving from energy tech adoption=0.8 |
|  | Provide more knowledge to implement sustainable behaviours and practices through newspaper, TV advertisements, and posters. (80% increase in implementation strength) | A2 | perceived fraction of water saving given water tech adoption=0.9; perceived fraction of energy saving from energy tech adoption=0.9 |
|  | Provide more knowledge to implement sustainable behaviours and practices through newspaper, TV advertisements, and posters. (100% increase in implementation strength) | A3 | perceived fraction of water saving given water tech adoption=1; perceived fraction of energy saving from energy tech adoption=1 |
| Reduce daily washing length | At present, the average daily washing length is about 4 minutes in Napoli. | BAU | initial duration of water use per capita [washbasin]=4 |
|  | Reduce the average daily washing length by 20%. | B1 | initial duration of water use per capita [washbasin]=3.2 |
|  | Reduce the average daily washing length by 40%. | B2 | initial duration of water use per capita [washbasin]=2.4 |
|  | Reduce the average daily washing length by 60%. | B3 | initial duration of water use per capita [washbasin]=1.6 |
| Reduce shower frequency | At present, the average shower frequency is about 0.57/cap/day. | BAU | average frequency of water use per capita per day= (0.57,8,5,1,0.21,6) |
|  | Reduce the shower frequency by 20%. The average shower frequency will be 0.46/cap/day. | B4 | average frequency of water use per capita per day= (0.46,8,5,1,0.21,6) |
|  | Reduce the shower frequency by 40%. The average shower frequency will be 0.34/cap/day. | B5 | average frequency of water use per capita per day= (0.34,8,5,1,0.21,6) |
|  | Reduce the shower frequency by 60%. The average shower frequency will be 0.23/cap/day. | B6 | average frequency of water use per capita per day= (0.23,8,5,1,0.21,6) |
| Decrease meat and dairy consumption | At present, the average fraction of meat & dairy consumption is about 35% in Napoli. | BAU | initial fraction of food choice adoption[meat and dairy]=0.35;  initial fraction of food choice adoption in 2010[nonmeat and nondairy]=0.65 |
|  | Reduce the fraction of meat & dairy consumption by about 5% on the current basis. The average fraction of meat & dairy consumption will be 0.3. | B7 | initial fraction of food choice adoption[meat and dairy]=0.3;  initial fraction of food choice adoption[nonmeat and nondairy]=0.7 |
|  | Reduce the fraction of meat & dairy consumption by about 10% on the current basis. The average fraction of meat & dairy consumption will be 0.25. | B8 | initial fraction of food choice adoption[meat and dairy]=0.25;  initial fraction of food choice adoption[nonmeat and nondairy]=0.75 |
|  | Reduce the fraction of meat & dairy consumption by about 15% on the current basis. The average fraction of meat & dairy consumption will be 0.2. | B9 | initial fraction of food choice adoption[meat and dairy]=0.2;  initial fraction of food choice adoption [nonmeat and nondairy]=0.8 |
| Mass information provision to household about the benefit of sustainable behaviour | Current situation with respect to sustainable behaviours in Napoli. There is still great potential for the promotion of sustainable behaviours in Napoli. | BAU | perceived fraction of water saving from water behaviour adoption=0.5; perceived fraction of energy saving from energy behaviour adoption=0.5; perceived fraction of nonmeat and nondairy health and environment benefit=0.5 |
|  | Provide more knowledge of implementing sustainable behaviours and practices through newspaper, TV advertisements, and posters. (60% increase in implementation strength) | B10 | perceived fraction of water saving from water behaviour adoption=0.8; perceived fraction of energy saving from energy behaviour adoption=0.8; perceived fraction of nonmeat and nondairy health and environment benefit=0.8 |
|  | Provide more knowledge to implement sustainable behaviours and practices through newspaper, TV advertisements, and posters. (80% increase in implementation strength) | B11 | perceived fraction of water saving from water behaviour adoption=0.9; perceived fraction of energy saving from energy behaviour adoption=0.9; perceived fraction of nonmeat and nondairy health and environment benefit=0.9 |
|  | Provide more knowledge to implement sustainable behaviours and practices through newspaper, TV advertisements, and posters. (100% increase in implementation strength) | B12 | perceived fraction of water saving from water behaviour adoption=1; perceived fraction of energy saving from energy behaviour adoption=1; perceived fraction of nonmeat and nondairy health and environment benefit=1 |
| Information provision for household to maintain sustainable consumption behaviour currented adopted | At present, the average maintenance of resource curtailment habits is about 1 year. One year later, the residents will abandon the adopted sustainable behaviour gradually. | BAU | maintenance duration of water saving behaviour=1; maintenance duration of energy saving behaviour=1 |
|  | Increase the maintenance of resource curtailment habits by 100%, which means the average maintenance of resource saving habits being increased to 2 years. | B13 | maintenance duration of water saving behaviour=2; maintenance duration of energy saving behaviour=2 |
|  | Increase the maintenance of resource curtailment habits by 200%, which means the average maintenance of resource saving habits being increased to 3 years. | B14 | maintenance duration of water saving behaviour=3; maintenance duration of energy saving behaviour=3 |
|  | Increase the maintenance of resource curtailment habits by 300%, which means the average maintenance of resource saving habits being increased to 4 years. | B15 | maintenance duration of water saving behaviour=4; maintenance duration of energy saving behaviour=4 |
| Increase the unit price of gas | At present, the unit price of gas is 0.188 Euro/m^3^. | BAU | average gas price rate=0.188 |
|  | Increase the unit price of gas by 40%. The unit price of gas will be 0.263 Euro/m^3^ after increase. | P1 | average gas price rate=0.188*(1+40%) |
|  | Increase the unit price of gas by 60%. The unit price of gas will be 0.301 Euro/ m^3^ after increase. | P2 | average gas price rate=0.188*(1+60%) |
|  | Increase the unit price of gas by 80%. The unit price of gas will be 0.338 Euro/ m^3^ after increase. | P3 | average gas price rate=0.188*(1+80%) |
|  | Increase the unit price of gas by 100%.The unit price of gas will be 0.376 Euro/ m^3^ after increase. | P4 | average gas price rate=0.188*(1+100%) |
| Increase the unit price of electricity | At present, the unit price of electricity is 0.337 Euro/kWh. | BAU | average electricity price rate=0.037 |
|  | Increase the unit price of electricity by 40%. The unit price of electricity will be 0.4718 Euro/kWh after increase. | P5 | average electricity price rate=0.037*(1+40%) |
|  | Increase the unit price of electricity by 60%. The unit price of electricity will be 0.5392 Euro/kWh after increase. | P6 | average electricity price rate=0.037*(1+60%) |
|  | Increase the unit price of electricity by 80%. The unit price of electricity will be 0.6066 Euro/kWh after increase. | P7 | average electricity price rate=0.037*(1+80%) |
|  | Increase the unit price of electricity by 100%. The unit price of electricity will be 0.674 Euro/kWh after increase. | P8 | average electricity price rate=0.037*(1+100%) |
| Increase the unit price of water | At present, the unit price of water is 1.13 Euro/ m^3^. | BAU | average water price rate=0.00113 |
|  | Increase the unit price of water by 40%. The unit price of water will be 1.582 Euro/m^3^ after increase. | P9 | average water price rate=0.00113*(1+40%) |
|  | Increase the unit price of water by 60%. The unit price of water will be 1.808 Euro/m^3^ after increase. | P10 | average water price rate=0.00113*(1+60%) |
|  | Increase the unit price of water by 80%. The unit price of water will be 2.034 Euro/m^3^ after increase. | P11 | average water price rate=0.00113*(1+80%) |
|  | Increase the unit price of water by 100%. The unit price of water will be 2.26 Euro/m^3^ after increase. | P12 | average water price rate=0.00113*(1+100%) |
| Increase the unit price of meat & dairy | At present, the unit price of meat and dairy is about 0.0074 Euro/g. | BAU | average meat and dairy price rate=0.0074 |
|  | Increase the unit price of meat & dairy by 40%. The unit price of meat & dairy will be 0.01036 Euro/g after increase. | P13 | average meat and dairy price rate=0.0074*(1+40%) |
|  | Increase the unit price of meat & dairy by 60%. The unit price of meat & dairy will be 0.0118 Euro/g after increase. | P14 | average meat and dairy price rate=0.0074*(1+60%) |
|  | Increase the unit price of meat & dairy by 80%. The unit price of meat & dairy will be 0.0133 Euro/g after increase. | P15 | average meat and dairy price rate=0.0074*(1+80%) |
|  | Increase the unit price of meat & dairy by 100%. The unit price of meat & dairy will be 0.0148 Euro/g after increase. | P16 | average meat and dairy price rate=0.0074*(1+100%) |

**S3. Results of field data survey**

| **Question** | | **Mean value** | **Measure unit** |
| --- | --- | --- | --- |
| How many persons are there in your house? | | 4 | N/A |
| How many showers do you take in a week? | | 4 | N/A |
| How long does each shower take? | | 4 | minutes |
| How many times does your family wash dishes in a day? | | 4 | N/A |
| How long does the water run each time? | | 8 | minutes |
| How many times does your family run the dishwasher a week? | | 3 | N/A |
| How long is each load? | | 50 | minutes |
| How many washing-machine loads does your family do each week? | | 6 | N/A |
| How long is each load? | | 60 | minutes |
| How many times does your family flush the toilet per day? | | 5 | N/A |
| How many times do you use the faucet for washing hands or tooth brush? | | 6 | N/A |
| How long does the water run each wash? | | 4 | minutes |
| Flow rate of your water-using appliances | Shower | 5 | L/min |
|  | Toilet | 4 | L/flush |
|  | Faucet in kitchen | 6 | L/min |
|  | Washing machine | 1 | L/load |
|  | Dish washer | 1 | L/load |
|  | Faucet in bathroom | 3 | L/min |
| Time of use for energy-using appliances | Lights | 6 | Hours/day |
|  | Heating/air conditioning | 5 | Hours/day |
|  | TV, computer and other electronic appliances | 12 | Hours/day |

1. **Water-saving behaviours**

| **Choice & Reasons** | **Yes. I always do the water saving actions** | | **No. I never do this.** | |
| --- | --- | --- | --- | --- |
|  | Environmental concern. *Awareness of water crisis and environmental protection* | Economic benefits. *Less expenditure from water saving* | Perceived inconvenience. *Discomfort from water saving* | Other reasons |
| Turning off the water when soaping up in the shower | 87% | 13% | 0% | 0% |
| Turning off the water when brushing teeth | 94% | 6% | 0% | 0% |

1. **Water-saving appliances**

| **Choice & Reasons** | **Yes. I already did or I will do this.** | | **No. I never do this.** | |
| --- | --- | --- | --- | --- |
|  | Environmental concern. *Awareness of water crisis and environmental protection* | Economic benefits. *Less expenditure from water saving* | Perceived inconvenience. *Perceived time-wasted from water-saving appliances installation/Discomfort from water-saving appliances installation* | Economic concern. *The water-saving appliances are expensive* |
| Installing the low flow tap and showerhead | 60% | 27% | 0% | 13% |
| Installing the low volume and dual flush toilet | 53% | 27% | 0% | 20% |
| Using water-efficient washing machine/ dishwasher | 67% | 27% | 0% | 6% |

1. **Energy-saving behaviours**

| **Choice & Reasons** | **Yes. I already did or I will do this.** | | **No. I never do this.** | |
| --- | --- | --- | --- | --- |
|  | Environmental concern. *Awareness of energy crisis and environmental protection* | Economic benefits. *Less expenditure from energy saving* | Perceived inconvenience. *Discomfort from energy saving* | Other reasons |
| Turning off the lights when unnecessary | 80% | 20% | 0% | 0% |
| Turning the heating down or off when you go out for a few hours or when you go to bed at night | 53% | 40% | 7% | 0% |
| Unplugging the electronic appliances when not in use | 67% | 27% | 6% | 0% |
| Lowering your water heater temperature. | 60% | 33% | 7% | 0% |

1. **Energy-saving technologies**

| **Choice & Reasons** | **Yes. I already did or I will do this.** | | **No. I never do this.** | |
| --- | --- | --- | --- | --- |
|  | Environmental concern. *Awareness of energy crisis and environmental protection* | Economic benefits. *Less expenditure from energy saving* | Perceived inconvenience. *Perceived time-waste from energy-saving appliances installation/ Discomfort from energy-saving appliances installation* | Economic concern. *The energy-saving appliances are expensive* |
| Installing energy-efficient water heater | 60% | 33% | 7% | 0% |
| Using energy-efficient light bulbs | 73% | 27% | 0% | 0% |
| Installing energy-efficient refrigerator | 60% | 40% | 0% | 0% |
| Installing energy-efficient electronic heater or air conditioner | 54% | 40% | 0% | 6% |
| Installing energy-efficient electronic appliances | 67% | 33% | 0% | 0% |

1. **Food choices**

| **Choice & Reasons** | **I’d like to choose nonmeat and nondairy foods for my diet** | | | **I prefer to include meat & dairy foods for my diet** |
| --- | --- | --- | --- | --- |
|  | Environmental concern. *The meat & dairy foods have higher carbon footprint.* | Economic reason. *It’s cheaper than meat & dairy foods* | Health benefits. *It benefits my health* |  |
| Your choice | 27% | 0% | 6% | 67% |

1. **Water & electricity & natural gas bill per month**

| **Item** | **Mean bill cost** | **Price unit** | **Item** | **Mean price rate** | **Price unit** |
| --- | --- | --- | --- | --- | --- |
| *Water bill per month* | 40 | € | *Water price rate* | 1.13 | €/m^3^ |
| *Electricity bill per month* | 100 | € | *Electricity price rate* | 0.037 | €/kWh |
| *Natural gas bill per month* | 40 | € | *Natural gas price rate* | 0.188 | €/m^3^ |

**S4. Simulation results**

|  | **NO.** | **Year** | **2020** | | | **2035** | | | **2050** | | |
| --- | --- | --- | --- | --- | --- | --- | --- | --- | --- | --- | --- |
|  |  | **Scenarios description** | **Electricity** | **Gas** | **Water** | **Electricity** | **Gas** | **Water** | **Electricity** | **Gas** | **Water** |
| B | BE1 | Provide more knowledge of adopting resource-saving appliances through newspaper, TV advertisements, and posters. (60% increase in implementation strength) | 0.05% | 0.06% | 0.15% | 0.07% | 0.06% | 0.23% | 0.05% | 0.07% | 0.20% |
|  | BE2 | Provide more knowledge of adopting resource-saving appliances through newspaper, TV advertisements, and posters. (80% increase in implementation strength) | 0.05% | 0.06% | 0.18% | 0.10% | 0.06% | 0.31% | 0.07% | 0.07% | 0.28% |
|  | BE3 | Provide more knowledge of adopting resource-saving appliances through newspaper, TV advertisements, and posters. (100% increase in implementation strength) | 0.05% | 0.06% | 0.26% | 0.10% | 0.13% | 0.42% | 0.07% | 0.07% | 0.36% |
|  | BE4 | Provide more knowledge of implementing sustainable behaviours and practices through newspaper, TV advertisements, and posters. (60% increase in implementation strength). | 0.83% | 0.32% | 0.22% | 0.87% | 0.39% | 0.23% | 0.90% | 0.39% | 0.20% |
|  | BE5 | Provide more knowledge of implementing sustainable behaviours and practices through newspaper, TV advertisements, and posters. (80% increase in implementation strength). | 1.07% | 0.39% | 0.29% | 1.14% | 0.45% | 0.27% | 1.15% | 0.46% | 0.28% |
|  | BE6 | Provide more knowledge of implementing sustainable behaviours and practices through newspaper, TV advertisements, and posters. (100% increase in implementation strength). | 1.30% | 0.52% | 0.37% | 1.38% | 0.58% | 0.35% | 1.39% | 0.59% | 0.36% |
|  | BG1 | Reduce the average daily washing length by 20%. | 1.25% | 2.45% | 9.17% | 1.26% | 2.33% | 9.16% | 1.22% | 2.28% | 9.16% |
|  | BG2 | Reduce the average daily washing length by 40%. | 2.48% | 4.90% | 18.34% | 2.49% | 4.73% | 18.36% | 2.44% | 4.56% | 18.37% |
|  | BG3 | Reduce the average daily washing length by 60%. | 3.73% | 7.35% | 27.55% | 3.73% | 7.06% | 27.56% | 3.69% | 6.78% | 27.57% |
|  | BG4 | Reduce the shower frequency by 20%. The average shower frequency will be 0.46/cap/day. | 0.16% | 0.64% | 1.10% | 0.17% | 0.58% | 1.08% | 0.15% | 0.59% | 1.08% |
|  | BG5 | Reduce the shower frequency by 40%. The average shower frequency will be 0.34/cap/day. | 0.32% | 1.23% | 2.28% | 0.34% | 1.23% | 2.27% | 0.32% | 1.17% | 2.28% |
|  | BG6 | Reduce the shower frequency by 60%. The average shower frequency will be 0.23/cap/day. | 0.46% | 1.81% | 3.39% | 0.48% | 1.75% | 3.39% | 0.45% | 1.69% | 3.40% |
|  | BG7 | Reduce the fraction of meat & dairy consumption by about 5 % on the current basis. The average fraction of meat & dairy consumption will be 0.3. | 0.21% | 0.06% | 0.04% | 0.29% | 0.06% | 0.00% | 0.30% | 0.07% | 0.00% |
|  | BG8 | Reduce the fraction of meat & dairy consumption by about 10 % on the current basis. The average fraction of meat & dairy consumption will be 0.25. | 0.44% | 0.13% | 0.04% | 0.65% | 0.13% | 0.04% | 0.67% | 0.13% | 0.04% |
|  | BG9 | Reduce the fraction of meat & dairy consumption by about 15% on the current basis. The average fraction of meat & dairy consumption will be 0.2. | 0.72% | 0.19% | 0.04% | 1.07% | 0.26% | 0.04% | 1.12% | 0.26% | 0.04% |
|  | BG10 | Increase the maintenance of resource curtailment habits to 2 years. | 0.19% | 0.45% | 1.44% | 0.19% | 0.45% | 1.42% | 0.17% | 0.39% | 1.44% |
|  | BG11 | Increase the maintenance of resource curtailment habits to 3 years. | 0.26% | 0.58% | 1.99% | 0.27% | 0.58% | 2.00% | 0.25% | 0.52% | 2.00% |
|  | BG12 | Increase the maintenance of resource curtailment habits to 4 years. | 0.30% | 0.64% | 2.28% | 0.31% | 0.65% | 2.27% | 0.30% | 0.59% | 2.28% |
| P | P1 | Increase average gas price rate by 40%. | 0.21% | 0.13% | 0.00% | 0.24% | 0.19% | 0.00% | 0.20% | 0.20% | 0.00% |
|  | P2 | Increase average gas price rate by 60%. | 0.30% | 0.19% | 0.00% | 0.34% | 0.26% | 0.00% | 0.30% | 0.26% | 0.00% |
|  | P3 | Increase average gas price rate by 80%. | 0.42% | 0.26% | 0.00% | 0.46% | 0.32% | 0.00% | 0.40% | 0.33% | 0.00% |
|  | P4 | Increase average gas price rate by 100%. | 0.51% | 0.26% | 0.00% | 0.56% | 0.39% | 0.00% | 0.52% | 0.39% | 0.00% |
|  | P5 | Increase average electricity price rate by 40%. | 0.12% | 0.06% | 0.00% | 0.12% | 0.13% | 0.00% | 0.10% | 0.13% | 0.00% |
|  | P6 | Increase average electricity price rate by 60%. | 0.16% | 0.13% | 0.00% | 0.19% | 0.13% | 0.00% | 0.15% | 0.13% | 0.00% |
|  | P7 | Increase average electricity price rate by 80%. | 0.23% | 0.13% | 0.00% | 0.24% | 0.19% | 0.00% | 0.20% | 0.20% | 0.00% |
|  | P8 | Increase average electricity price rate by 100%. | 0.28% | 0.19% | 0.00% | 0.29% | 0.19% | 0.00% | 0.25% | 0.20% | 0.00% |
|  | P9 | Increase average water price rate by 40%. | 0.07% | 0.13% | 0.63% | 0.12% | 0.19% | 0.85% | 0.07% | 0.13% | 0.76% |
|  | P10 | Increase average water price rate by 60%. | 0.12% | 0.19% | 0.85% | 0.17% | 0.26% | 1.19% | 0.12% | 0.20% | 1.04% |
|  | P11 | Increase average water price rate by 80%. | 0.14% | 0.19% | 1.03% | 0.19% | 0.26% | 1.42% | 0.15% | 0.20% | 1.20% |
|  | P12 | Increase average water price rate by 100%. | 0.14% | 0.26% | 1.14% | 0.22% | 0.32% | 1.58% | 0.15% | 0.20% | 1.32% |
|  | P13 | Increase average meat and dairy price rate by 40%. | 0.00% | 0.00% | 0.00% | 0.00% | 0.00% | 0.00% | 0.00% | 0.00% | 0.00% |
|  | P14 | Increase average meat and dairy price rate by 60%. | 0.00% | 0.00% | 0.00% | 0.00% | 0.00% | 0.00% | 0.00% | 0.00% | 0.00% |
|  | P15 | Increase average meat and dairy price rate by 80%. | 0.00% | 0.00% | 0.00% | 0.00% | 0.00% | 0.00% | 0.00% | 0.00% | 0.00% |
|  | P16 | Increase average meat and dairy price rate by 100%. | 0.00% | 0.00% | 0.00% | 0.00% | 0.00% | 0.00% | 0.00% | 0.00% | 0.00% |
|  |  |  |  |  |  |  |  |  |  |  |  |
|  |  |  |  |  |  |  |  |  |  |  |  |
|  |  |  | | | | | | | |  |  |
|  |  |  |  |  |  |  |  |  |  |  |  |
|  | **Polcy impact** | **Weak** |  |  |  |  |  |  | **Strong** |  |  |
|  |  |  |  |  |  |  |  |  |  |  |  |


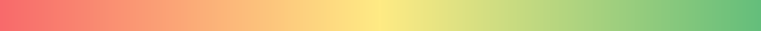


**S5. Sensitivity analysis**

|  | **Year** | **2020** | | | **2035** | | | **2050** | | |
| --- | --- | --- | --- | --- | --- | --- | --- | --- | --- | --- |
|  | **Scenarios description** | **Electricity use** | **Gas use** | **Water use** | **Electricity use** | **Gas use** | **Water use** | **Electricity use** | **Gas use** | **Water use** |
| Cost (water tech environment/energy tech environment/water behaviour environment/energy behaviour environment/food environment ) | Cost-20% | 0.175% | 0.182% | 0.110% | 0.204% | 0.182% | 0.154% | 0.186% | 0.183% | 0.160% |
|  | Cost-10% | 0.088% | 0.061% | 0.074% | 0.113% | 0.061% | 0.077% | 0.093% | 0.061% | 0.080% |
|  | Cost+10% | -0.066% | -0.061% | -0.037% | -0.090% | -0.121% | -0.077% | -0.093% | -0.061% | -0.080% |
|  | Cost+20% | -0.132% | -0.121% | -0.074% | -0.181% | -0.182% | -0.154% | -0.186% | -0.122% | -0.160% |
| Environment (water tech cost/energy tech cost/water behaviour cost/energy behaviour cost/food cost) | Environment-20% | -0.986% | -0.546% | -0.184% | -1.109% | -0.728% | -0.231% | -1.093% | -0.671% | -0.200% |
|  | Environment-10% | -0.482% | -0.243% | -0.074% | -0.520% | -0.364% | -0.115% | -0.535% | -0.305% | -0.120% |
|  | Environment+10% | 0.460% | 0.304% | 0.074% | 0.498% | 0.303% | 0.077% | 0.488% | 0.305% | 0.080% |
|  | Environment+20% | 0.899% | 0.486% | 0.147% | 0.973% | 0.606% | 0.154% | 0.930% | 0.610% | 0.120% |
|  |  |  |  |  |  |  |  |  |  |  |
|  |  | 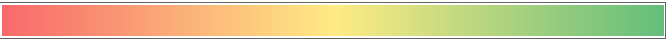 | | | | | | |  |  |
|  |  |  |  |  |  |  |  |  |  |  |
|  |  | **weak** |  |  |  |  |  |  | **strong** |  |

**References**

1. Sterman JD. Business dynamics: systems thinking and modeling for a complex world. Nachdr. Boston: Irwin/McGraw-Hill; 2009.

2. Urban J, Ščasný M. Structure of Domestic Energy Saving: How Many Dimensions? Environ Behav. 2016;48: 454–481. doi:10.1177/0013916514547081

3. R. (Rizky) J. System Dynamics Model for FEW (Food-Energy-Water) Nexus in the Netherlands. TU Delft; 2018. doi:10.4121/UUID:11717773-EBB6-401E-BB15-3EC8430A6BCB

4. Hussien WA, Memon FA, Savic DA. An integrated model to evaluate water-energy-food nexus at a household scale. Environ Model Softw. 2017;93: 366–380. doi:10.1016/j.envsoft.2017.03.034
