# Supplementary material for: Simulations of scenarios for urban household water and energy consumption
Source: PLoS One. 2021 Apr 7;16(4):e0249781. doi: 10.1371/journal.pone.0249781 (PMC8026023; doi:10.1371/journal.pone.0249781)
Supplement: Attachment — Submitted filename: NEXUS.pdf [file pone.0249781.s002.pdf]

The authors claimed that previous methods of assessing resource consumption and delivering of suitable policies for the conservation of these key resources were limited. This is because apart from utilizing only one specific resources (food, water, and energy) at household level and employing food-water-energy (FEW) nexus approach among urban dwellers, household behavioral preferences were largely excluded in assessing resource consumption scenarios. To overcome the previous limitations, the authors proposed a novel approach based on household scenarios for food, water and energy interlinkages and incorporating individual preference behavior in these scenarios, chosen metropolitan city of Naples as a case study. Grounded on earlier casual-loop structure, the proposed household FEW nexus model was divided into four interface: that is food, water, energy consumption and the combined FEW nexus, also interaction between them were depicted in casual loop diagrams. The combined effect of these casual loop systems culminated into household level scenarios for consumption of electricity, gas and water by dwellers in Naples city. Also, the action of individual behavioral preference towards the conservation of these key resources were simulated. To help policy makers, the authors considered the fact that electricity, gas and water are the key resources among urban dwellers, conducted simulation of household scenarios targeting policy-oriented strategies, such as price strategies and conservation strategies in their study.

The proposed method was applied to the metropolitan city of Naples (Southern Italy). The authors obtained comprehensive data from official sources and also carried out a survey of urban dwellers, using 248 university students as volunteer residents in metropolitan city of Naples to support their study.

The findings and the results indicated that the percentage of electricity, gas and water use are related to different scenarios with respect to BAU scenarios, that is they are sensitive with respect to initial household's preferences whereas the discussion explores the use of such scenarios to improve the awareness of household behavior impact on environmental resources conservation for informed policy action. This of course is vitally important to urban households planning committees.

The manuscript with the title Simulation of scenarios for urban household water and energy consumption is suitable for publication in PLOS one journal. The following comments should be address in the manuscript

1. There are a lot of grammatical errors in the manuscript
2. The legends in Figures 4, 5 and 6 should be made brighter
3. In organization of paper, authors only bolded Sections, I suggest that the subsections should also be bolded but different font size.
