# Supplementary material for: Simulations of scenarios for urban household water and energy consumption
Source: PLoS One. 2021 Apr 7;16(4):e0249781. doi: 10.1371/journal.pone.0249781 (PMC8026023; doi:10.1371/journal.pone.0249781)
Supplement: Attachment — Submitted filename: Response_Urban_household_resouces_REV01.pdf [file pone.0249781.s003.pdf]

Response to Reviewers

Simulations of scenarios for urban household water and energy consumption

Dear Editor,
Dear Reviewers,

Thank you very much for your efforts and work to support us in improving the quality of our manuscript.

Introductory reply

We agree with all the indications given by the reviewers. We noticed the presence of several grammar errors. In order to remove them and to improve the quality of the manuscript, we required the support of AJE professional editing services, being one of the companies indicated by PLOS ONE website. In particular, the manuscript was edited for proper English language (British English), grammar, punctuation, spelling, and overall style.

Following the professional editing indication, we modified the title from "Simulation of scenarios for urban household water and energy consumption" to "Simulations of scenarios for urban household water and energy consumption".

Besides the language revision, the quality editing identified 1 error in Figure 5 (the lack of separation between "Time" and "(year)"), which was corrected. The figure originally appeared to be Figure 7 (before moving Figure 1 and Figure 2 to the Supplementary Materials file, numbering them as Figure 1S and Figure 2S). The words "Non meat & diary" was modified by the editing service as "Nonmeat and nondairy". Since the same text was reported in Figure 2 (now, Figure 2S), we revised the figure accordingly.

Following the indications given by Reviewer #1, we moved part of the method, related to details about the simulator structure, in the supplementary materials file, section S1. In fact, we believe that this suggested option enhances the readability of the manuscript. Consequently, we re-numbered the Supplementary Material file sections accordingly.

Following the indications given by Reviewer #2, we improved the brightness of the legends in Figures 4, 5 and 6 (now, Figures 2, 3 and 4). Besides the sub-section titles size (originally 12 pt, now 11 pt), the style review by AJE detected some inconsistencies in the main text (sometimes, the main text was 12 pt, while, in other parts, the text size was 11 pt). We modified the main text size, using a uniform 11 pt size.

Figure 1 and Figure 2 are now numbered as Figure 1S and Figure 2S, since they are now found in the Supplementary Materials file. Consequently, the numbering of figures has changed.

Figures 4, 5 and 6 are now Figures 2, 3 and 4. All figures numbers have been changed and checked. The same check has been performed along the text to have the correct relation between the main text and figures.

Detailed response to reviewers

Reviewer #1:

1. Introduction: It is good, but it lacks a comprehensive setup. Authors could consider revising it as it is not linked up nicely especially the first paragraph seems more like in bits and pieces.

Thank you for your comment. The poor language quality made the text not linked up nicely, as you state here. This is why we asked a language revision, also including a style revision.

2. Methods and Results. Technically strong and well explained. However, I could have condensed the methods with an explanation given in the supplementary text (Just a suggestion).

Thank you for your comment. Thinking about you suggestion, we decided to move the sub-section "Household FEW nexus structure" to the supplementary materials file, section S1. In fact, we believe that this suggested option enhances the readability of the manuscript. Consequently, we added a sentence in the main text to indicate the presence of section S1. We also re-numbered the following supplementary materials sections accordingly.
Figure 1 and Figure 2 are now numbered as Figure 1S and Figure 2S, since they are now found in the Supplementary Materials file. Consequently, the figures numbering has changed. Figures 4, 5 and 6 are now Figures 2, 3 and 4.

Reviewer #2

1. There are a lot of grammatical errors in the manuscript

We agree with your comment. In order to remove them and to improve the quality of the manuscript, we required the support of AJE professional editing services, being one of the companies indicated by PLOS ONE website. In particular, the manuscript was edited for proper English language (British English), grammar, punctuation, spelling, and overall style.

2. The legends in Figures 4, 5 and 6 should be made brighter

We agree with your comment. We modified the legends of the three figures accordingly.
Please, note that Figure 1 and Figure 2 are now numbered as Figure 1S and Figure 2S, since they are now found in the Supplementary Materials file. Consequently, the numbering of figures has changed. In particular, Figures 4, 5 and 6 are now Figures 2, 3 and 4. All figures numbers have been changed and checked. The same check has been performed along the text to have the correct relation between the main text and figures.

3. In organization of paper, authors only bolded Sections, I suggest that the subsections should also be bolded but different font size.

We agree with your comment. We noticed the problem. We modified the text in the following

way:
Section title: Times New Roman, bold, 12 pt
Sub-section title: Times New Roman, bold, 11 pt
Main text: Times New Roman, normal, 11 pt
Figures and tables captions: Times New Roman, normal, 10 pt

Final remarks

We include the professional revision certificate together with the reviews files.

Finally, we hope that you will appreciate our efforts in improving the manuscript quality, following your requirements and suggestions.

AJE®

Editing Certificate

This document certifies that the manuscript

Simulation of scenarios for urban household water and energy consumption

prepared by the authors

Marco Casazza, Jingyan Xue, Shupan Du, Gengyuan Liu, Sergio Ulgiati

was edited for proper English language, grammar, punctuation, spelling, and overall style by one or more of the highly qualified native English speaking editors at AJE.

This certificate was issued on **February 26, 2021** and may be verified on the AJE website using the verification code **112A-875B-12B2-3E7F-5D81** .

AJE® EDITING CERTIFIED

Neither the research content nor the authors' intentions were altered in any way during the editing process. Documents receiving this certification should be English-ready for publication; however, the author has the ability to accept or reject our suggestions and changes. To verify the final AJE edited version, please visit our verification page at aje.com/certificate. If you have any questions or concerns about this edited document, please contact AJE at support@aje.com.

AJE provides a range of editing, translation, and manuscript services for researchers and publishers around the world.
For more information about our company, services, and partner discounts, please visit aje.com.
