# Supplementary material for: Simulations of scenarios for urban household water and energy consumption
Source: PLoS One. 2021 Apr 7;16(4):e0249781. doi: 10.1371/journal.pone.0249781 (PMC8026023; doi:10.1371/journal.pone.0249781)
Supplement: Attachment — Submitted filename: PLOS ONE 1.pdf [file pone.0249781.s004.pdf]

The current version of the manuscript is a significant improvement over the previous one. All the concerns I raised in the previous manuscript have been addressed by the authors. Therefore, without any reservation, the manuscript should be considered for publication.
